# Evaluation of Genotype MTBDRsl Assay to Detect Drug Resistance Associated with Fluoroquinolones, Aminoglycosides and Ethambutol on Clinical Sediments

**DOI:** 10.1371/journal.pone.0049433

**Published:** 2012-11-15

**Authors:** Kanchan Ajbani, Chaitali Nikam, Mubin Kazi, Christen Gray, Catharina Boehme, Kavita Balan, Anjali Shetty, Camilla Rodrigues

**Affiliations:** 1 Department of Microbiology, P. D. Hinduja Hospital and Medical Research Centre, Veer Sarvarkar Marg, Mahim, Mumbai, India; 2 Foundation for Innovative New Diagnostics (FIND), Geneva, Switzerland; University of Cape Town, South Africa

## Abstract

**Background:**

The emergence of resistant tuberculosis (TB) is a major setback to the global control of the disease as the treatment of such resistance is complex and expensive. Use of direct detection of mutations by molecular methods could facilitate rapid diagnosis of resistance to offset diagnostic delays. We evaluated the performance of the Genotype MTBDRsl (Hain Life Sciences) for the detection of second line resistant TB directly from stored smear positive sputum sediments.

**Methodology/Principal Findings:**

The assay showed a diverse range of sensitivity and specificity, 91.26% [95% CI, 84–96] and 95.5% [95% CI, 87–99] for FQ (PPV ∼97% & NPV ∼ 87.67%), 56.19% [95%CI, 46–66] and 81% [95%CI, 66–91] for EMB (PPV ∼ 88.06% & NPV ∼ 43.21%) and 100% for SLD. Diagnostic accuracy for FQ, SLD and EMB was 94%, 100% and 63.51%, respectively. 1.17% (2/170) were heteroresistance strains, where the heteroresistance was linked to *rrs* gene. A varying rate of validity was observed 100% (170/170) for FQ, 94.11% (160/170) for EMB, 88.23% (150/170) for SLD.

**Conclusions/Significance:**

Genotype MTBDRsl is simple, rapid, economical assay that can be used to detect commonly known resistance associated with Fluoroquinolone, second line injectable drugs and ethambutol. The assay detects the targeted resistance in less time as compared to phenotypic DST. But due to low NPV to FQ (88%) and EMB (43.21%), the assay results must be interpreted in coordination with the phenotypic DST.

## Introduction

The most critical components of TB control are prompt identification and rapid implementation of effective treatment regimen to curtail transmission. Inadequate treatment regimen can select for drug resistant organism (acquired resistance) and transmission of these resistant bugs can lead to primary resistance in individuals. [1]

The emergence of multi drug resistant TB (MDR- TB) {resistance to isoniazid (INH and rifampicin (RIF)} and extensively drug resistant TB (XDR- TB) {MDR- TB as well as additional resistance to any fluoroquinolone (FQ) and second line injectable drugs such as Kanamycin(KAN), Amikacin(AM) and capreomycin(CAP)} threatens the efforts to reduce the global burden of TB. [2]

The diagnosis of TB still relies heavily on the conventional methods of culture identification and drug susceptibility (DST) wherein culture takes about 3–6 weeks and DST in liquid medium takes about additional 10–12 days. [3] Inappropriate treatment regimen during the period till the DST results are available may result in increase of resistance and further transmission of this resistance compounds the issue.

The DST for SLD is complex, time consuming and costly. Instead use of direct rapid molecular technique to detect resistance through presence of mutations can be a great tool to decrease the diagnostic delay. The Genotype MTBDR plus assay has shown to have a good sensitivity and specificity for prediction of RIF and INH resistance both from direct sputum specimens and culture isolates. In 2008, World Health Organization (WHO) has recommended the use of the Genotype MTBDR plus kit for the detection of RIF resistance from smear positive clinical specimens. [4].

Hain Life Sciences have developed another kit Genotype MTBDRsl which detects resistance to FQ (by targeting the commonly known mutations in the QRDR in gyrase A region) [5], SLD (by targeting the commonly known 1401 and 1484 mutations in the rrs gene) [6] and ethambutol (EMB) (by targeting the *emb* 306 mutation in the emb gene). [7] We evaluated the performance of this Genotype MTBDRsl kit with 170 smear positive clinical sputum specimens. The results were compared with the phenotypic DST done in Mycobacterial growth indicator tube (MGIT 960) (BD BioScience) using the WHO recommended critical concentrations. [8].

## Materials and Methods

### Setting

This study was carried out at P. D. Hinduja National Hospital and Medical Research centre (PDHNH) a tertiary care hospital in Mumbai, India.

#### Ethical approval

Waiver of consent was received from IRB committee.

### Clinical (sputum) Sediments

A total of 170 sediments were collected between Feb 2011–Oct 2011 with 74 specimens having 3+smear status, 50 with 2+and 46 with 1+(graded as per WHO recommended criteria) [9]. DST testing at our laboratory is performed only on request from the treating physician/clinician. PDHNH being a tertiary care centre receives cases that are either treatment failure or relapse or MDR/XDR suspects. Acid fast bacilli (AFB) smear 1+positive (Light microscopy was used to read the smears) 170 sediments were selected consecutively from those where phenotypic DST was performed thus having a referral bias towards nonresponders. The selection pressure on MTB to develop resistance is strong and the patient population at our centre is different from elsewhere in the country.

### Sample Processing

2 ml of sputum sample was processed by NALC-NaOH method, as described elsewhere. [10] Finally the sediment was resuspended in 2 ml of PBS (pH = 7.4). Of this 500 µl was inoculated in 7 ml MGIT tube and 500 µl was inoculated on solid media (LJ). The sediment was transferred to 2 ml screw cap tubes and was preserved at −80°C. Sample sediments were thawed as required.

### DST Procedure

All isolates were tested by conventional DST by using MGIT 960 for FQ (ofloxacin and moxifloxacin), second line injectables (amikacin, capreomycin) and EMB using critical concentrations recommended by WHO. [8] Among the SLD kanamycin was tested at a critical concentration of 5 µgm/ml [11]. The drug powders were procured from Sigma laboratories except for moxifloxacin and ethambutol which were procured from Cipla and Becton Dickinson respectively.

For Quality Control on a routine weekly basis a known genotypic confirmed resistant strain was put up for DST in MGIT 960 and H37Rv as the pan susceptible strain. Biannual proficiency testing is performed as per College of American Pathologists (CAP) guidelines by sequencing five resistant and susceptible isolates for the hot spot regions for each of the SLD.

### Genotype MTBDR sl Assay

The procedure was divided into 3 steps:

- DNA extraction: 500 microlitre of decontaminated sediment is centrifuged at 14,000 rpm for 15minutes; this is resuspended in 100 microlitre of sterile distilled water. This was further processed with heat lysis and sonication.
- Mulitplex polymerase chain reaction (PCR) amplification with biotinylated primers: PCR was performed in Eppendorff thermal cyclers with the following cycling conditions, Denaturation 95°C/15 min, initial Denaturation 950 C/30 sec, Annealing 58°C/2 min,(10 cycles) Denaturation 95°C/25 sec, Annealing 53°C/40 sec, Extension 70°C/40 sec (30 cycles), Final Extension 70°C/8 min.
- Reverse Hybridisation was performed as per the manufacturer’s instructions.

The laboratory follows a strict unidirectional work flow. For the Quality Control measures on the line probe with each batch that was run a known pan susceptible strain H37Rv and a known genotypically confirmed strain with known mutations for the second line was also tested. A negative control was also added with each batch so as to ensure no cross contamination has occurred.

### MTBDRsl Strip

Each strip of the assay has a total of 22 reaction zone [12]. These are as follows **1**. Conjugate control **(CC)** indicates efficiency of conjugate binding and substrate binding. **2** Amplification control **(AC)** indicates the efficiency of PCR, a missing AC band indicates the test to be repeated. **3**. *M. Tuberculosis* **(TUB)** band presence indicates of *M. Tuberculosis complex*. 4. Locus Control (LC) for each of the target (*gyrA, rrs, embB*) – detects gene region specific for respective locus. 5 *gyrA* WT1 (85–90), *gyrA* WT2 (89–93), *gyrA* WT3 (92–97), *gyrA* MUT1 (A90V), *gyrA* MUT2 (S91P), gyrA MUT3A (D94A), gyrA MUT3B (D94N/Y), gyrA MUT3C (D94G), gyrA MUT3D (D94H). 6. rrs WT1 (1401–1402), rrs (1484), rrs MUT1 (A1401G), rrs MUT2 (G1484T) 7. emb WT(306), emb MUT 1A(M306I), emb MUT 1B (M306V). [12].

Results for MTBDRsl assay are interpreted based on the hybridization (presence of sharp visible band) to the respective probes coated on to the strips. A test is valid and interpretable. when Conjugate controls (CC), Amplification control (AC) and M. tuberculosis complex (TUB) bands are visible along with gyrA Locus control (LC), rrs LC, emb LC; absence of any one of the band makes the test invalid/indeterminate for the respective target. In addition, presence of wild type sequence along with the corresponding mutant probe indicates the sample carrying heteroresistance strain.

### Sequencing

Equal number of representative discrepant sediments of phenotypic resistance and genotypic susceptible as well as phenotypic susceptible and genotypic resistance would be sequenced.

### Stastical Analysis

Sensitivity, specificity, positive predicative value (PPV), Negative predicative Value (NPV) was calculated in comparison to MGIT phenotypic DST (gold standard). Metadisc software (Ver1.4) was used to calculate the Sensitivity, specificity, positive predicative value (PPV), Negative predicative Value (NPV).

## Results

### Phenotypic DST

Of the 170 sediments analyzed 77.64% (132/170) are MDR (INH+RIF)^ R^ of these 15.9% (21/132) are XDR, 5.88% (10/170) were monoresistant to isoniazid and susceptible to rifampicin, ofloxacin, moxifloxacin and second line aminoglycosides tested (kanamycin, amikacin and capreomycin). Three (1.7%) were resistant to only ofloxacin and moxifloxacin but susceptible to the other drugs tested, a single isolate was resistant to isoniazid as well as fluoroquinolones but susceptible to other drugs. **12.9%** (22/170) were susceptible to all drugs tested.

#### Phenotypic FQ-DST

Of the total 170 sample sediments 99 isolates were resistant to ofloxacin and moxifloxacin, 4 isolates were resistant to ofloxacin alone. Three phenotypic sensitive strains detected as resistant by the Genotype MTBDRsl assay; of these one sample was revived from contaminated specimen, which on repeat DST continued to be susceptible. The other two specimens were repeated for phenotypic DST and it showed resistance correlating with the assay.

Thus after the resolution of the discrepancies, there were 101 strains resistant to both ofloxacin and moxifloxacin and 65 strains were susceptible to both. [Table 1].

**Table 1:** Phenotypic DST results.

N = 170	FQ	KAN, AM, CAP	Emb
**Resistant [R]**	101	16	114
**Sensitive [S]**	65	148	56
Mono resistance*	4	6	0

Monoresistance in FQ is seen as resistant to ofloxacin.

#### Phenotypic second line aminoglycosides DST

Of the total 170 sediments, 148 isolates were susceptible to KAN, AM, CAP; 16 isolates were resistant to KAN, AM, CAP and 6 were resistant to KAN and AM but sensitive to CAP. [Table 1].

#### Phenotypic DST-EMB

Out of 170 sediments 114 isolates were resistance to EMB and 56 susceptible. [Table 1].

### Genotype MTBDRsl Assay

**Figure 1:**
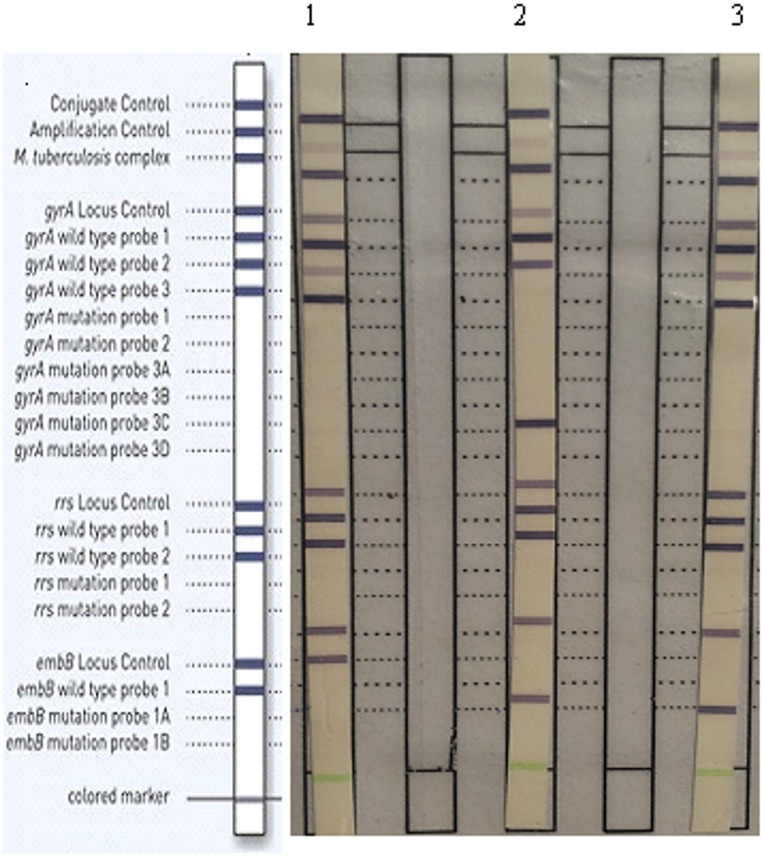
Representative DNA patterns obtained with GenoType MTBDR*sl*. The positions of the oligonucleotides and control probes are given on the left. The targeted genes and specific probes lines are shown from top to bottom as follows: conjugate control (CC); amplification control (AC); *M. tuberculosis* complex-specific control (TUB); *gyrA* amplification control; *gyrA* wild-type probes WT1 to WT3 (85–90, 89–93 and 92–97); *gyrA* mutant probes MUT1, MUT2, MUT3A, MUT3B, MUT3C, and MUT3D for codons A90V, S91P, D94A, D94N, D94Y, D94G, and D94H, respectively; *rrs* amplification control; *rrs* wild-type probes WT1 (codons 1401 and 1402) and WT2 (codon 1484); *rrs* mutant probes MUT1 and MUT2, with A1401G and G1484T changes, respectively; *embB* amplification control; *embB* wild-type probe WT1, covering codon 306; and *embB* probes MUT1A and MUT1B for the mutations M306I and M306V, respectively. Lane 1, example of an fluoroquinolone, second line aminoglycoside and ethambutol susceptible; lane 2, fluoroquinolone resistance due to mut 3C, aminoglycosides susceptible and ethambutol resistance due to *embB* mutant M306V; lane 3, fluoroquinolone and aminoglycoside susceptible and ethambutol resistance with M306V.

Of all the sediments tested 88.23% (150/170) samples were found valid to all the three targets *gyrA* (FQ), *rrs* (SLD), *embB* (EMB) respectively by Genotype MTBDRsl assay, the remaining 11.76% (20/170) were found invalid/indeterminate to SLD or EMB by the assay either due to absence of rrs bands alone [figure 1].

### Genotype MTBDRsl Assay FQ Results

Out of 105 FQ phenotypic- resistant strains 89 (90%) strains were directly identified as resistant by MTBDRsl assay by hybridization to mutant probes [Table 2]; two isolates (2.02%) showed double pattern of mutation and 3 (3%) isolates were indirectly identified as resistant by lack of hybridization with WT1 and WT3 and no hybridization to any of the mutant probe. [Table 2] The 4 monoresistant to ofloxacin showed hybridisation with MUT1 i.e. A90V. Three phenotypic- sensitive strains were detected as resistance by the assay. Of these one isolate was revived from contaminated specimen that on repeat DST continued to be susceptible. The other two were repeated for phenotypic DST and it showed resistance to both ofloxacin and moxifloxacin correlating with the Genotype MTBDRsl assay. These 3 isolates were sequenced and found to be correlating with the Genotype MTBDRsl assay. In contrast, **9** phenotypic- resistant (both ofloxacin and moxifloxacin) strains were detected as sensitive by the assay as the test detects resistance originating in gyrA QRDA region, resistance originating from mutations of other gene/gene region is not detected by the assay. A representative isolates phenotypically resistant to fluoroquinolones were sequenced for the gyrA hot spot region and no mutations were found.

**Table 2:** Genotypic gyrA pattern obtained by MTBDRsl assay on 170 clinical sediments.

PhenotypicDST	Codon mutation	No of Isolates	%
**R**	D94G	41	42.26% (41/97)
	A90V	23	23.71% (23/97)
	D94Y/N	11	11.3% (11/97)
	D94A	11	11.3% (11/97)
	S91P	6	6.18% (6/97)
	D94N/Y+D94G	1	1.03% (1/97)
	A90V+D94G	1	1.11% (1/97)
	ΔWT1	2	2.06% (2/97)
	ΔWT3	1	1.11% (1/97)
**S**	**WT1+WT2+WT3**	**64**	
	**Indeterminate**	**Nil**	**Nil**

ΔWT = lack of hybridization.

Of the 65 phenotypic- sensitive strains 64 (98.4%) were correctly identified as sensitive by MTBDRsl. [Table 3] The concordance between phenotypic test and MTBDRsl assay was 89.52% (94/105) for detecting FQ resistant.

**Table 3:** Genotype MTBDRsl assay analysis in comparison to phenotypic DST.

		Culture – DST					
		FQ*		KAN, AM, CAP		EMB	
		R	S	R	S	R	S
**Genotype** **MTBDRsl assay**	**R**	96	1	22	0	60	8
	**S**	9	64	0	128	46	36

* The FQ results are calculated after the resolution of discrepancies.

### Genotype MTBDRsl Assay Second Line Injectables Results

16 sediments resistant to second line injectables were correctly identified by MTBDRsl assay as resistant; by hybridization to one of the mutant rrs probe MUT1 indicating mutation in the region from 1400 to 1500 of the rrs gene that confers resistant to injectables. Additionally, 6 sediments that were CAP sensitive but resistant to KAN, AM were found to be resistant; hybridization at MUT1.

128 sediments susceptible to second line injectables were correctly identified as susceptible by the MTBDRsl assay as none of it showed hybridization with MUT1 or MUT 2, but showed hybridization at WT1 and WT2. The concordance between phenotypic test and MTBDRsl assay was 100% (22/22) for detecting injectable resistant [Table 3]. However, 11.76% (20/170) showed indeterminate results by the assay due to absence of rrs bands i.e. no amplification or insufficient amplification probably due to low copy number of rrs gene (n = 1) per MTB genome. [18].

### Genotype MTBDRsl Assay EMB Results

Among 114 phenotypic- resistant strains 59 (51.7%) were correctly identified as resistant by MTBDRsl assay by hybridization to the mutant probes M306V and M306I [Table 4]. Out of 56 phenotypic susceptible strains 35 (62.5%) were correctly identified as susceptible to EMB by the assay.

**Table 4:** Genotypic emb pattern obtained by MTBDRsl assay on 170 clinical sediments.

Phenotypic DST	Codon mutation	No of Isolates	%
**R**	M306I	19	25.67% (19/74)
	M306V	56	75.6% (56/74)
**S**	**WT1**	**85**	
	**Indeterminate**	**10**	**5.88%(10/170)**

#### Sensitivity and specificity of the assay

The statistical values for the assay were calculated by comparing the assay results with phenotypic DST using MGIT 960. The overall sensitivity of MTBDRsl assay to detect resistant to different drugs is as follows: 100% for second line injectables, 91% [95% CI, 84–96] for fluoroquinolones with PPV ∼ 99% and NPV ∼ 88% and 56.2% [95% CI, 46–66] for ethambutol with PPV ∼88.06% and NPV ∼43.21%. [Table 3, 5]. The specificity of MTBDRsl assay to detect susceptibility to different drugs is as follows 100% for SLD, 98% [95% CI, 87–99] for fluoroquinolones and 81% [95% CI, 66–91] for ethambutol.

**Table 5:** Statistical summary of GenoTypeMTBDR*sl assay.*

	FQ	SLD	EMB
**Sensitivity**	91%	100%	56.19
**Specificity**	98%	100%	81%
**Positive predict Value (PPV)**	99%	1	88.06%
**Negative predict Value (NPV)**	88%	1	43.21%
**Prevalence**	62%	14.67%	61%
**Diagnostic Accuracy**	94%	100%	63.51%

95.23% (20/21) concordance was observed in detection of XDR cases. A single XDR strain [4.7% (1/21)] which was phenotypically FQ resistant was detected as FQ sensitive by the assay (confirmed by sequencing) but SLD and EMB resistant both by phenotypic as well as by MTBDRsl assay might be due to mutation other than gyrA.

## Discussion

With overall increase in the incidence of resistant TB [2], a rapid molecular test is the need of the hour as compared to conventional drug susceptibility tests which are time consuming laborious and cumbersome. GenoType MTBDR*sl* is NAT-based molecular, single test assay for the simultaneous detection of *M. tuberculosis* (MTB) complex and its resistance to FQ, second line injectables, and EMB. The assay has been designed to detect the presence of most frequent mutation found in *gyrA, rrs, embB* gene that confers resistance to FQ, second line injectables, and EMB respectively. The current study evaluates the performance of genotype MTBDRsl assay on smear positive sputum sediments (n = 170). A few studies have been performed worldwide to evaluate the performance of genotype MTBDRsl assay directly on to the clinical samples with a sample size of 64 (Germany) [13], 59 (Italy) [14] and 54 (Spain) [15] where the overall rate of a valid test/indeterminate test was 93.7% (60/64)/3.5% (4/64), 89.3% (53/59)/10.16% (6/59) and 92.5% (50/54)/7.4%(4/54). [13], [14], [15].

In the present study, the overall rate for reporting a valid test was 88.23% (150/170) [100% FQ (*gyrA*) (170/170), 94.11% EMB (*embB*) (160/170) and 88.23% Second line injectables (*rrs*)] and the rate for indeterminate test was 11.76% (20/170) which is in an acceptable range in comparison to the above mentioned studies [13], [14], [15]. Different studies carried out either on clinical isolate or on clinical sediment demonstrated similar sensitivity and specificity ranging from 70–90% for FQ [16], and 80–100% [17] for Second line injectables and 55–77% [18] for EMB which implies there is slight to no difference in detecting resistance when using clinical sediment as a starting material for the assay. Our results showed a variable sensitivity to different drugs (100% second line injectables, 91% FQ & 56.19% EMB), and specificity (100% second line injectables, 98% FQ & 81.9% EMB), respectively, which is in an acceptable range in comparison to other studies performed using clinical isolate or clinical sediment [13], [14], [15], [17], [19], selected consecutively from those where phenotypic DST was performed thus having a referral bias towards nonresponders. The selection pressure on MTB to develop resistance is strong and the patient population at our centre is different from elsewhere in the country.

Among the gyrA targeted mutants in the assay D94G was the frequently observed mutation [42.26% (41/97)], followed by A90V 23.71% (23/97), D94Y/N, D94A [11.3%(11/97)] and S91P [6.18% (6/97)], which is in agreement to other studies [17]. In our study no mutations related to D94H have been detected, a rare in-silico designed mutant, which is in concordance to other studies [18], however one study [17] reported a single isolate with D94H mutant. In addition, 8.7% (9/103) phenotypic resistant strains were detected as sensitive by the assay as there are several fluoroquinolone resistance mechanisms in mycobacteria besides mutations in the *gyrA* (60–90%) [20] and gyrB genes, like parC, parE, qnr genes [21] and enhancement of efflux pumps etc. [5] As the present assay only detects mutations in QRDR region of gyrA, 9 strains were undetected by the assay. Hence, sensitivity by the assay must be interpreted in combination with additional laboratory and clinical data. The overall sensitivity and specificity for second line aminoglycosides was 100% (the number of sediments resistant to second line aminoglycosides is relatively small) which is high in comparison to other studies, where the sensitivity was in the range of 70–100% and specificity was 100%. Mutation in A1401G is related to high level resistance to second line aminoglycosides [6], [22]. In this study, A1401G [100% (22/22)] was the only reported mutation. 11.76% (20/170) showed indeterminate results due to lack of hybridization to rrs probe which might be due to presence of inhibitor or low copy of rrs gene (n = 1) [22] per MTB genome. Furthermore, 1.17% (2/170) sediments were detected as heteroresistant. The heteroresistance was in association to *rrs* gene/target, where as previous studies have reported heteroresistance mostly in relation to *gyrA* target. In order to decrease the rate of indeterminate tests, samples should be ideally processed immediately after decontamination.

The sensitivity and specificity for detecting ethambutol was 56.19% and 81% which is very low as compared to FQ and second line injectables, but is in concordance to other studies [13], [17]. The most common mutation detected by the assay was M306V 74.32% (55/74) followed by M306I 25.67% (9/74) which is high in case of M306V as compared to previous studies. Other mutations are required to be targeted [23] by the assay to increase its sensitivity and specificity. It has been observed that genotypic analysis identified high rate of mutations (91.4%) at codon 306 of the *embB* gene in comparison to phenotypic analysis, where phenotypic test failed to identify EMB resistance [24]. But 40% (46/115) of the EMB resistance cases were not detected by the assay, which is same as reported in the previous study. In the current scenario, detection of EMB resistance by targeting mutations at 306 codon has a mixed opinion by different authors which is due to poorer inter-laboratory performance for EMB than some other drugs, and it has been suggested that discrepancies between genotypic and phenotypic testing may be due to difficulties with phenotypic testing [23], [25], [26].

FQ and second line injectables are resorted to be used in treatment of cases that are treatment failures, relapses or MDR/XDR-TB suspects. Detection of resistance to FQ and second line injectables by conventional method (a two step process) takes approximately 15–30 days to report DST results, due to slow growing nature of *M. tuberculosis* [13], [27]. The time required to detect resistance by MTBDR*sl* is 1–2 days after receiving the sample.

Although, there are numerous molecular based test like Multiplex allele specific (MAS-PCR) [28], Reverse Line Blot Hybridization (RLBH) [29], Hetero duplex analysis [30] that targets *gyrA, rrs, embB* to detect resistance, but to confirm the amplified product might require sequencing facility as reference standard to detect the mutation in coordination to above mentioned technique. Unfortunately, not all laboratories are equipped with sequencing facility as it is expensive and requires modern expertise.

In this study 21 strains were XDR, of which MTBDRsl was able to detect 95.23% (20/21) of cases but a single XDR strain was detected as FQ-sensitive, but resistant to Second line injectable and EMB by the assay, as the resistant to this strain might be due to other resistant mechanism [5] as there was no mutation detected by sequencing of the hot spot region. The limitation of the study is it included smear positive sputum samples only.

In conclusion, to break the chain of ongoing transmission a rapid molecular method, like MTBDRsl, would limit or decrease the rate of transmission by early detection of the resistance. Although the assay does not replace the phenotypic DST it is helpful in rapid detection of drug resistance among resistant suspects.

## References

[1] Mistry NF, Iyer AM, D’souza DT, Taylor M, Young DB, et al (2002) Spoligotyping of *Mycobacterium tuberculosis* Isolates from Multiple-Drug-Resistant Tuberculosis Patients from Bombay, India. Journal Of Clinical Microbiology, 40 (7): 2677–2680. 10.1128/JCM.40.7.2677-2680.2002 PMC120599 12089307

[2] Jassal M, Bishai WR (2009) Extensively drug-resistant tuberculosis, Lancet Infect Diseases. 9: 19–30. 10.1016/S1473-3099(08)70260-3 18990610

[3] Parsons LM, Somoskovi A, Gutierrez C, Lee E, Paramasivan CN, et al (2011) Laboratory Diagnosis of Tuberculosis in Resource –Poor countries: Challenges and Opportunities. Clinical Microbiology Reviews 24(2): 314–350. 21482728 10.1128/CMR.00059-10 PMC3122496

[4] World Health Orginisation. Policy guideline: Molecular Line Probe Assays for Rapid Screening of Patients of Multidrug resistant tuberculosis. Geneva: World Health Orginisation, 2008. Available: www.who.int/tb/laboratory/line_probe_assays/en. Accessed: July 05 2012.

[5] Maruri F, Sterling TR, Kaiga AW, Blackman A, Van der Heijden YF, et al (2012) A systematic review of gyrase mutations associated with fluoroquinolone-resistant *Mycobacterium tuberculosis* and a proposed gyrase numbering system. Journal of Antimicrob Chemother 67(4): 819–831. 22279180 10.1093/jac/dkr566 PMC3299416

[6] Jugheli L, Bzekalava N, Rijk P, Fissette K, Portaels F, et al (2009) High Level of Cross-Resistance between Kanamycin, Amikacin, and Capreomycin among *Mycobacterium tuberculosis* Isolates from *Georgia and a Close Relation* with Mutations in the *rrs* Gene. Antimicrobial Agents And Chemotherapy, 53 (12): 5064–5068. 10.1128/AAC.00851-09 PMC2786337 19752274

[7] Zhang Y, Yew WW (2009) Mechanisms of drug resistance in *Mycobacterium tuberculosis.* . Int J Tuberc Lung Dis 13(11): 1320–1330. 19861002

[8] World Health Orginisation (2008) Policy guideline on drug susceptibility testing (DST) of second line antimicrobial drugs Geneva: World Health Orginisation, 2008.

[9] World Health Organization (1997) Treatment of Tuberculosis: Guidelines for national programmes, 2nd ed. WHO/TB/97.220, Geneva, 1997.

[10] Rodrigues C, Almedia D, Shenai S, Goyal N, Mehta A (2007) Dedicated decontamination: A necessity to prevent cross-contamination in High throughput Mycobacteriology Laboratories. Indian Journal of Medical Microbiology, 25(1), 4–6. 10.4103/0255-0857.31053 17377344

[11] Rodrigues C, Jani J, Shenai S, Thakkar P, Siddiqi S, et al (2008) Drug susceptibility testing of *Mycobacterium tuberculosis* against second-line drugs using the Bactec MGIT 960 System. International Journal of tuberculosis and Lung diseases 12(12): 1449–1455. 19017456

[12] Hain Life science Kit inserts for MTBDRsl (Version 1), IFU: 3XX-XX., Available: www.hain-lifescience.de/en/Accessed: July 05 2012.

[13] Hillemann D, Rüsch-Gerdes S, Richter E (2009) Feasibility of the GenoType MTBDR*sl* Assay for Fluoroquinolone, Amikacin-Capreomycin, and Ethambutol Resistance Testing of *Mycobacterium tuberculosis* Strains and Clinical Specimens. Journal Of Clinical Microbiology 47(6): 1767–1772. 19386845 10.1128/JCM.00081-09 PMC2691112

[14] Miotto P, Cabibbe A, Mantegani P, Borroni E, Fattorini L, et al.. (2012) Genotype MTBDRsl performance on clinical samples with diverse genetic background. European respiratory journal, In press. 10.1183/09031936.00164111 22267773

[15] Lacoma A, Garcia-Sierra N, Part C, Maldonado J, Ruiz-Manzano J, et al (2012) Genotype MTBDrsl for molecular detection of second line drug and ethambutol resistance in *Mycobacterium tuberculosis.* . Journal of Clinical Microbiology, 50 (1): 30–36. 10.1128/JCM.05274-11 PMC3256720 22075597

[16] Ignatyeva O, Kontsevaya I, Kovalyov A, Balabanova Y, Nikolayevskyy V, et al. (2012) Detection of resistance to second-line antituberculosis drugs using the Genotype® MTBDR*sl* assay: a multi-center evaluation and feasibility study. J. Clin. Microbiol., In press. 10.1128/JCM.00039-12 PMC3347138 22378910

[17] Brossier F, Veziris N, Aubry A, Jarlier V, Sougakoff W (2010) Detection by genotype MTBDR*sl* Test of Complex Mechanisms of Resistance to Second-Line Drugs and Ethambutol in Multidrug- Resistant *Mycobacterium tuberculosis* Complex Isolate. Journal Of Clinical Microbiology 48(5): 1683–1689. 20335420 10.1128/JCM.01947-09 PMC2863904

[18] Huang WL, Chi TL, Wu MH, Jou R (2011) Performance Assessment of the GenoType MTBDR*sl* Test and DNA Sequencing for Detection of Second-Line and Ethambutol Drug Resistance among Patients Infected with Multidrug-Resistant *Mycobacterium tuberculosis.* . Journal Of Clinical Microbiology 49(7): 2502–2508. 21562102 10.1128/JCM.00197-11 PMC3147822

[19] Kiet V, Lan NT, An DD, Dung NH, Hoa DV, et al (2010) Evaluation Of The MTBDRsl Test For Detection Of Second-Line-Drug Resistance In *Mycobacterium Tuberculosis.* . Journal Of Clinical Microbiology, 48 (8): 2934–2939. 10.1128/JCM.00201-10 PMC2916598 20573868

[20] Chakravorty S, Aladegbami B, Thomas K, Lee JS, Lee EG (2011) Rapid detection of Fluroquinolone-Resistant and Heteroresistant Mycobacterium tuberculosis by use of Sloppy Molecular Becons and Dual Melting-Temperature Codes in a Real Time PCR assay. Journal of Clinical Microbiology 49(3): 932–940. 21191047 10.1128/JCM.02271-10 PMC3067712

[21] Shen GH, Tsao TC, Kao SJ, Lee JJ, Chen YH, et al (2012) Does empirical treatment of community-acquired pneumonia with fluoroquinolones delay tuberculosis treatment and result in fluoroquinolone resistance in *Mycobacterium tuberculosis*? Controversies and solutions. International Journal of Antimicrobial Agents 39(3): 201–205. 22285045 10.1016/j.ijantimicag.2011.11.014 PMC7127649

[22] Alangaden G, Kreiswirth B, Aouad A, Khetarpal M, Igno F, et al (1998) Mechanism of Resistance to Amikacin and Kanamycin in *Mycobacterium tuberculosis.* . Antimicrobial Agents And Chemotherapy 42(5): 1295–1297. 9593173 10.1128/aac.42.5.1295 PMC105813

[23] Shrivastava S, Garg A, Ayyagari A, Nyati KK, Dhole TN, et al (2006) Nucleotide polymorphism associated with ethambutol resistance in clinical isolates of *Mycobacterium tuberculosis* . Current microbiology 53: 401–405. 16972132 10.1007/s00284-006-0135-1

[24] Johnson R, Jordaan AM, Pretorius L, Engelke E, Van dar Spuy G, et al (2006) Ethambutol resistance testing by mutation detection. Int J Tuberc Lung Dis 10(1): 68–73. 16466040

[25] Plinke C, Cox HS, Kalon S, Doshetov D, Rüsch-Gerdes S, et al (2009) Tuberculosis ethambutol resistance: Concordance between phenotypic and genotypic test results. Tuberculosis 89: 448–452. 19800845 10.1016/j.tube.2009.09.001

[26] Vasanthi N, Natarajan A, Manupriya S, Muthuraj M, Usharani B, et al (2010) Characterization of *Emb CB* Genes Associated with Ethambutol Resistance in Human Isolates of *Mycobacterium tuberculosis.* . International Journal of Microbiological Research 1(1): 14–21.

[27] Piersimoni C, Olivieri A, Benacchio L, Scarparo C (2006) Current Perspectives on Drug Susceptibility Testing of *Mycobacterium tuberculosis* Complex: the Automated Nonradiometric Systems. Journal of Clinical Microbiology, 44 (1): 20–28. 10.1128/JCM.44.1.20-28.2006 PMC1351976 16390942

[28] Vadwai V, Shetty A, Rodrigues C (2012) Multiplex allele specific PCR for rapid detection of extensively drug resistant tuberculosis. Tuberculosis, In press. 10.1016/j.tube.2012.01.004 22342856

[29] Ajbani K, Shetty A, Mehta A, Rodrigues C (2011) Rapid diagnosis of Extensively Drug Resistant tuberculosis using a Reverse Line Blot Hybridization assay. Journal of Clinical Mirobiology 49(7): 2546–51. 10.1128/JCM.02511-10 PMC3147869 21613436

[30] Johnson R, Streicher E, Louw G, Warren RM, Van Helden PD, et al (2006) Drug Resistance in *Mycobacterium tuberculosis.* Curr. Issues Mol. Biol. 8: 97–112. 16878362

